# Enablers and Barriers of Blended Learning in Faculty Development

**DOI:** 10.7759/cureus.22853

**Published:** 2022-03-04

**Authors:** Yusuf Yilmaz, Halil Ibrahim Durak, Soner Yildirim

**Affiliations:** 1 Department of Medical Education, Faculty of Medicine, Ege University, Izmir, TUR; 2 Department of Computer Education and Instructional Technology, Middle East Technical University, Ankara, TUR

**Keywords:** multiple case study, online, enablers and barriers, qualitative study, faculty development, blended learning

## Abstract

Introduction

Online learning provides a ubiquitous and self-paced learning experience, while face-to-face learning encourages commitment in a prescheduled formal instruction. Blended learning (BL) combines these two mediums and provides flexible learning opportunities. While faculty development programs have utilized these two mediums separately, BL has not been fully implemented locally until recently. Identifying elements that enable or hinder faculty within a newly implemented BL program can enhance the learning experience and support professional development. The current study aims to identify how junior and senior faculty members of medical departments at a Turkish university perceive enablers and barriers in a new BL faculty development program.

Methods

This research is a multiple case study with qualitative inquiry using in-depth interviews and thematic analysis. Using a BL approach, the research team designed faculty development activities based on the Four-Component Instructional Design model. Participants accessed the activities on a Moodle learning management system. Faculty experiences in blended faculty development were examined. The study group consisted of 26 participants, with 14 junior faculty in case 1 and 12 senior faculty in case 2 from different medical departments at a Turkish university. Data were collected and analyzed using qualitative methods.

Results

This study identified enablers and barriers within a BL faculty development program. While participants identified three barriers, they identified eight enabling elements in a BL program. A lack of time was the most critical barrier to participation in the program. Setting goals for personal development and obtaining skills in teaching were essential enablers within the BL program.

Conclusion

The use of an online platform to support face-to-face faculty development programs is beneficial in several ways for faculty. Faculty developers can utilize BL to foster engagement and motivate faculty for increased participation, especially if they seek to mitigate known barriers to a successful BL program. Online communication and activities are suggested to develop communities of practice in the workplace. Strategies to eliminate workload and provide guidance on time management are required for both junior and senior faculty.

## Introduction

Faculty development has a key role in capacity building and establishing a growth mindset for faculty. Faculty development programs can foster skills and roles that are in demand. Faculty have reported positive attitudes toward these development programs in general [1] but indicated that the programs come with challenges [2-4]. Murray reported that the challenges for community college faculty consisted of a lack of goals and robust teaching methods, low faculty turn-out, and lack of evaluation [5]. Among medical faculty, the challenges of daily teaching and research activities are complicated by the additional requirements of clinical duties [6].

Various instructional approaches in faculty development have been conducted to overcome these challenges, such as workshops, seminars, short courses, and fellowship programs [7]. The approaches applied to faculty development programs have been successful, but each approach carries certain drawbacks or barriers. All these approaches employ face-to-face methods in which learners and faculty developers meet on fixed dates.

In addition to the conventional face-to-face methods, online learning options exist for faculty development, such as Communities of Practice (CoP) [2,8]. Online options better support long-term development goals [9]. CoP and other online options can ease the burden of faculty involvement in academic roles and facilitate this long-term involvement.

Blended learning (BL) combines two educational approaches, namely face-to-face and online, into one educational modality [10]. BL has the potential to mitigate negative aspects in both approaches [11]. To participate in face-to-face programming, participants and instructors must meet at a specific time and location. The time and location requirement of face-to-face can be a hindrance when participants and instructors have busy schedules and heavy workloads. Online learning eliminates the time and location logistical issues of face-to-face while also reducing costs and improving the quality of student learning [12]. However, a lack of social interaction can be detrimental to participation. BL has potential as a successful teaching modality for faculty learners and instructors by using beneficial elements of face-to-face and online modalities [2,13].

In a BL environment, it becomes essential to identify elements that act as barriers to learning and elements that facilitate learning to optimize the program's success and support professional development among faculty members. To identify these elements, qualitative research should be used that captures data from people in real-world conditions, as described by Yin [14]. This current study aimed to identify how junior and senior faculty members of medical departments at a Turkish university perceive enablers and barriers in a BL faculty development program.

## Materials and methods

Study design

This research used a multiple case study design with qualitative inquiry to understanding faculty experiences in a BL development program and is part of the Ph.D. thesis of the first author (Y.Y.) submitted to Middle East Technical University [14]. Using in-depth interviews and thematic analysis, we sought to examine the perception of faculty toward the BL faculty development program. The reason for selecting the multiple case study design is it allows comparison among cases. When studying descriptive or explanatory issues and attempting to gain a first-hand understanding of individuals and events, the case study method is a very effective methodology. The nature of BL in the current study creates a contemporary phenomenon. Moreover, “in-depth” and “real-life context” are other valid reasons to employ the methodology in the study. Faculty development programs are conducted during the participants’ work hours, along with their other duties of the clinician-educators such as patient care. Throughout the program, how faculty react to the program is a necessity since they should divide their working hours to cope with each of the roles their job demands of them. Two groups of participants enrolled in courses based on their medical career needs. One group (i.e., case 1) consisted of junior faculty members who had not previously received a faculty development certificate. Junior faculty case was defined in the study as an instructor or attending physician without faculty appointment, or faculty without a faculty development program certification. The second group (i.e., case 2) consisted of senior faculty. Senior faculty case was full professors or associate professors with faculty development certification.

Study context and participants

The current study took place at Ege University’s Faculty of Medicine. The Faculty of Medicine is a relatively large medical faculty by Turkish standards, with 43 departments contributing to the teaching activities of the faculty’s educational program for prospective doctors. There were 532 teaching faculty and attendings, 412 research assistants (i.e., residents), and 2,500 undergraduate medical students in the faculty [15]. The faculty’s hospital had a 1,816-bed capacity and serviced a total of 65,245 inpatients and 1,026,644 outpatients in 2015 [15].

Purposive convenience sampling was used in forming the study population, which consisted of participants in a faculty development program available from June 13, 2016, through July 1, 2016. From a potential pool of 20 junior faculty members, three did not consent to participate, and three could not attend the face-to-face sessions. Therefore, case 1 consisted of 14 participants.

Case 2 had 24 applicants; however, five subsequently withdrew their application, citing other urgent work. Of the remaining 19 applicants, five requested not to participate in the current research study, and two were unable to continue after the first course session. As a result, case 2 consisted of 12 participants.

Development and implementation of blended faculty development

**Figure 1:**
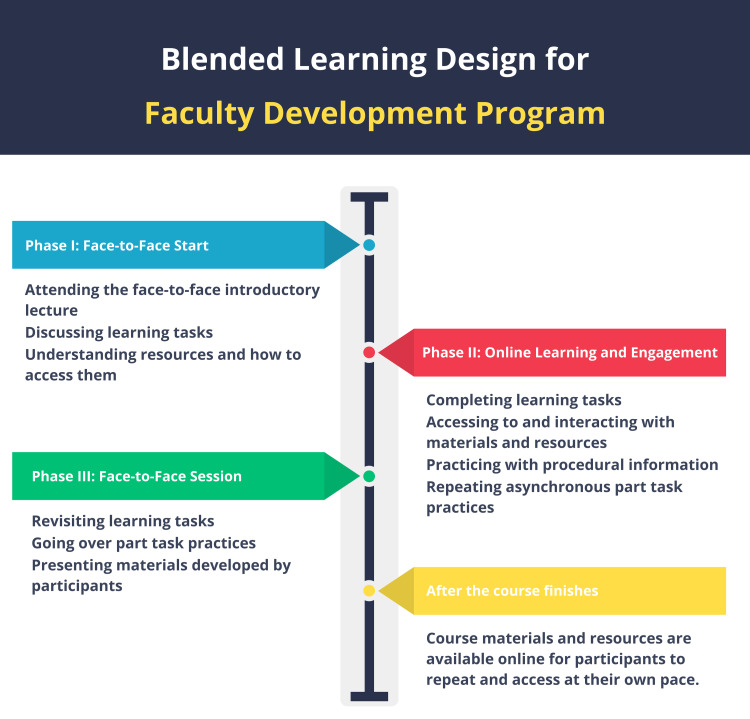
Blended learning design for the faculty development program

We developed a faculty development course to teach participants how to design and create online activities for their teaching activities. The faculty development course used the Four-Component Instructional Design (4C/ID) model [16]. The model consists of four components: learning tasks, supportive information, procedural information (i.e., just-in-time), and part-task practice. At all steps of the process, the primary researcher designed the BL faculty development course in consultation with experts. Each design step was evaluated and reviewed based on experts in medical education and instructional technology. Figure 1 shows the phases of the blended faculty development courses in the program.

The open-source learning platform Moodle (Moodle, West Perth, Australia) was used to deliver the online activities of the current study. As a part of the instructional design process, Moodle was enhanced with a mobile application to connect participants ubiquitously to reach the system quickly and access information when needed. Moreover, participants were notified about their course activities via mobile application notifications.

Ethics

The current study was conducted under the approval of the Human Subjects Ethics Committee of Middle East Technical University. All participants provided written informed consent to participate.

Data collection

An interview form was developed by the primary researcher of the current study to collect participant views on their experience in BL faculty development. The form consisted of basic questions to obtain the participant's gender, age, academic title, and experience with information communication technologies and BL, including a self-reported computer skill score using a scale of 1 to 5 points.

The form allowed a semi-structured interview to better understand a participant's views on blended faculty development courses. The final interview form consisted of 12 main questions and probing questions where necessary that covered the participants' experiences with BL faculty development courses.

We conducted in-depth semi-structured interviews within two weeks of the end of the program. The interviews took place in locations where the participants would feel comfortable, and the primary researcher conducted all interviews. Prior to each interview, participants were informed of the interview procedure. Participants provided consent to be audio-recorded, and the audio records were kept confidential and used only for this study. The interviews were conducted and analyzed in Turkish language and translated to English for reporting. Each participant's responses were masked and assigned a designation from P1 to P26.

Data analysis

We used the inductive method to analyze the interview data. Conversations were transcribed verbatim from the audio files, along with any notes. The average duration of the interviews was 43 minutes. NVivo software Version 11 (QSR International Inc., Burlington, MA) was used along with an audio playing feature to adjust the conversation speed and assign time codes to participant answers. Transcriptions were read and reread by the researcher to confirm their accuracy. Next, each interview transcription was coded in nodes. Emerging codes were aggregated in a master list of all codes from the interviews. The codes were merged into categories representing similar meanings. Each category was then labeled according to its theme.

Rigor and trustworthiness

Peer debriefing and member checking assured credibility within the study. The primary researcher consulted the other research team member supervisor and co-supervisor, and the assigned thesis monitoring committee during various stages of the study. Moreover, ideas and feedback from two independent scholars in medical education were sought throughout the study. The researcher also applied member checking during the interviews and after the transcription. In addition, two voluntary participants were invited to review the raw data and themes to eliminate issues caused by misinterpretation. No significant problems were raised during the volunteer participants' review meeting.

In the current study, several coders were invited at different stages of the analysis process. Before starting to code, each coder was informed about the research, the interviews, and the structure. The first coding round was conducted by the primary researcher and two coders, each of whom had a Ph.D. in healthcare. During the process, the coding structure and principal codes were described. Each coder then examined a small part of the data, discussing new emerging codes. The coders discussed the final codes and reached a consensus.

Confirmability is the exclusion of potential bias of the researcher in the reporting of a study's findings. The researchers played a significant part in the analysis, design, development, and implementation of the intervention of the current study into BL faculty development. The primary (Y.Y.) researcher holds both M.Sc. degrees in computer education and instructional technology and works as a lecturer in the medical education field. Senior researcher (S.Y.) is a full professor in Computer Education and Instructional Technology. H.I.D. is a full professor in the medical education field. All of these researchers have expertise in qualitative research.

## Results

The study included a total of 26 participants (16 females, 10 males) with a mean age of 41 years (standard deviation, SD: 6.9 years; range: 33 to 53 years). Table 1 presents participant demographic data. Participants had a mean self-reported computer skill score of 4.04 (SD: 0.77; range: 2 to 5).

**Table 1:** Demographic information of the participants

		Case 1	Case 2
		Percentage (n)	Percentage (n)
Academic title	Full professor	-	75% (9)
	Associate professor	36% (5)	25% (3)
	Assistant professor	7% (1)	-
	Attending physician	57% (8)	-
Department	Basic science	43% (6)	42% (5)
	Clinical medical science	36% (5)	50% (6)
	Surgical science	21% (3)	8% (1)
Faculty development program certified	No	86% (12)	17% (2)
	Yes	14% (2)	83% (10)
	Total	100% (14)	100% (12)
Computer skills self-assessment (M±SD)		4.1±0.7	4±0.9

Enablers of blended faculty development course

Among the enabling elements of BL for participants, eight themes emerged from the analysis. Table 2 presents the enabling themes and representative quotations of participants in cases 1 and 2, listed from most frequent to least frequent. For example, personal development was the most common enabling theme for case 1 participants, whereas improving teaching skills was the most common enabling theme for case 2 participants.

**Table 2:** Enabling themes and representative quotations for cases 1 and 2, presented from most frequent to least frequent f = code frequency

Case 1 (N=14)			Case 2 (N=12)		
Enabling Theme	n (f)	Case 1 Example Quotations	Enabling Theme	n (f)	Case 2 Example Quotations
Personal development	10 (14)	It becomes possible for the individual to see their shortcomings. I think it is the best one. Also, my tenure track is newly coming, these are the periods when we step into the associate professorship. Well, you know I did not have an account in entering question. The best is to being able to see shortcomings and then ask them to you. It is important to be able to get information from safe [trusted] people! P4	Improving teaching skills	5 (7)	I’m 40 years old, and there’s a huge generation difference between me and the new generation. I wanted to come to class to be able to tell them enough, more appropriate, and better-quality lessons. My main objectives were to offer more benefits to students and to learn how to use the Internet actively. P17
Improving teaching skills	8 (11)	At the moment, we are viewed as future faculty. From my point of view, I am getting the first steps of the education career. When I came across an education for a faculty, I thought I shouldn’t miss it and I was thrilled. I have to attend this education. For example, why do we have regular assistant trainings? Intern trainings are provided for second and third grade students. We become role model for the students. At this point, in order to get close to them, I came to learn the method of education while giving education. I can’t say that my qualifications were super when I came to the course, but I started to learn how to fish. Frankly, that was why I came, and I had motivation. P24	Job-related interest	4 (5)	I wanted to learn a lot and had this kind of experience when I was abroad. I was using another e-learning platform more actively. I wanted to create a platform of our own here and I was very motivated. P20
Certification	6 (11)	Actually, my purpose of getting this education was only to receive certificate. The reason of this was I had no information. Head of our department, our dean, mentioned that it would be useful if we attended and received this education, and this motivated me to participate. P9	Personal development	3 (4)	I have a mechanic for how it can be used in terms of my personal development. P14
Perceived quality of course	4 (4)	You will get rid of the useless information heap, and you will reach the information in short time from the target lecture notes showed by the faculty. Definitely it is something that will save time and the biggest benefit of it is that. P22	Incentives	2 (8)	Personal effort should be prioritized and supported with thanks. The biggest thing is to support lower base..., I’m more motivated when a letter of thanks comes. Sometimes the students, sometimes the dean, are sending me such letters. As a result, I’m quite happy. In the same way, a motivating letter of thanks can be sent from time to time as a result of participation and support in e-learning. P12
Learning climate in BL	3 (3)	After the technology is integrated, the socialization of people is increasing. The person cannot ask the question within 300 people but then they can ask via message. I think this situation is more advantageous in terms of self-improvement. Mutual interaction also has benefits in learning. You can learn something in front of the computer, but a humor or example that the teacher says at the moment can provide persistence. P7	Perceived quality of course	2 (2)	In classical system, everyone can use electronic technology very widely. For instance, while waiting for the flight on a trip, I can open my laptop or mobile device and get a chance to read. It’s really a timesaver. It is very important thing to our academic life. We might need to study everywhere. A guideline is published, we can immediately download and check it. The same thing can be provided to the students. We are already trying to provide now…however if you say this takes complete place of the lessons, I might disagree with. P1
Decrease time allocation for face-to-face sessions	1 (1)	There were friends of mine who couldn’t come to this program…the first thing they say that why it was 8 days. With the blended method, the reduction of face-to-face durations is good. P25	Certification	1 (1)	
Incentives	1 (1)	To create something tangible here. So, in the end, it’s one of its tasks. P25	Learning climate in BL	1 (1)	An interactive learning environment and self-direction of the faculty based on feedback are extremely important. P10
Job-related interest	1 (1)	Of course, I would enter the notes [I would upload to the system]. I might upload resources or motivating things. P25			

Personal Development

Personal development was the most common enabling theme in the BL experience for case 1 participants. Ten of 14 case 1 participants and three of 12 case 2 participants stated that the courses improved their abilities in areas they perceived as lacking in their competencies.

Improving Teaching Skills

Improving teaching skills was the most common enabling aspect for case 2 participants (five of 12) and was second most common for case 1 participants (eight of 12). As the current study's primary focus of the BL faculty development courses, improving teaching skills emerged from the analysis. In total, 13 (or half the study population) participants agreed that improving teaching skills was among the top enabling aspects of BL programs. Participants valued the scope of the courses and perceived the courses as an opportunity for professional development. Participant P24 stated their motivation for the courses was workplace responsibilities and possible knowledge to provide better teaching. Additionally, P17 stated their motivation was from a learner perspective, as improving their skills for the benefit of future generations motivated them to become a better educator.

Certification

Seven participants reported certification as an enabler (case 1, n=6; case 2, n=1). Certification as an enabling element was more common to case 1 participants than case 2 participants.

Perceived Quality of Course

The perceived quality of the course content was an essential element to the participants. As participant P22 mentioned, the courses were designed for immediate use in a short time. Participants perceived BL faculty development programming as effective use of their time as academics.

Learning Climate in BL

BL provided a learning environment where faculty could socialize in the face-to-face sessions. The online part of the learning was seen as supportive information, whereas knowledge retention was required to support the face-to-face sessions. Interaction and feedback were important aspects of BL.

Decrease Time Allocation for Face-to-Face Sessions

One participant mentioned the time allocation needed for attending face-to-face sessions as an enabling element. For long-duration courses, participation can be problematic, and courses can be hard to follow to completion. In this context, BL was seen as an approach that decreased the time required for face-to-face sessions.

Additional Incentives

One participant in case 1 and two in case 2 felt that additional incentives (beyond saving time, gaining knowledge, skills, and certification) were enabling elements for BL programs. Participants asked if attending the courses would be supported with anything additional, implying direct financial incentives in the interview transcripts.

Job-Related Interest

One participant in case 1 and four in case 2 stated that the potential for gaining knowledge and experience from the courses was an enabler for them. They perceived the BL approach as the current and future methodology of education and wanted to be part of its development. They felt that they could improve their skills and incorporate them into their teaching.

Barriers to blended faculty development course

Participant feedback on barriers within BL coalesced into three prevailing themes. Table 3 presents the common barrier themes and representative quotations of participants in cases 1 and 2, listed from most frequent to least frequent. Participants in both cases asserted the same themes but with different emphases. Case 1 participants reported lack of time as the most common barrier, while case 2 participants listed beliefs and assumptions as to the most common barrier.

**Table 3:** Barrier themes and representative quotations for cases 1 and 2, presented from most frequent to least frequent f = code frequency

Case 1 (N = 14)			Case 2 (N = 12)		
Barrier Theme	n (f)	Case 1 Example Quotations	Barrier Theme	n (f)	Case 1 Example Quotations
Lack of time	11 (16)	We had to continue because it was the last course however in background, we had a working environment that bothered us. I have to do this etc. After I left at 1 pm, I had to complete things within the office hours and because of this situation I had difficulties in completing the learning tasks, many people had. P9 For example, I came after I arranged all my shifts. We might not have been able to arrange. Because you have to arrange a lot of things consecutively. Coming in the morning can create a problem in many branches. P16	Beliefs and assumptions	7 (24)	Because we are traditional faculty member of a traditional school, just like I said, we get what we can get in the course, but it’s hard for us to do homework online. Because of that it comes up to that ratio [online and face-to-face balance]. Our culture is weak. P10. We are a nation who never read the manual of an electronic device. We are a nation who tinker with the devices. So, what can be done? For instance, in faculty development, homework can be asked from that videos. There should be indications like “Everyone will watch this video, and will do that” and it will appear that who has done what. There should be either stick or carrot for Turkish people, I am saying it including myself. Otherwise when we say “watch it we will meet tomorrow,” because there are no output of whether we watched or not, no one will not watch. P19
Beliefs and assumptions	6 (7)	After a certain age, the concentration decreases. P8. It is very important for the academician, I think we can adapt more; however, the adaptation of the older lecturers is much weaker. P16	Online learning abilities	5 (7)	People are afraid of also technological things. Technological things may need to be told a little more. If this is really wanted, the subsystem support can be established. P5
Online learning abilities	3 (4)	I had other projects in the evenings. I was restricted in there. I’ve a little hard time trying to find where to enter. There was a little deficiency in the information that guided me. That menu structure needs a little improvement. Today we even looked for 2-3 minutes to find out where to upload the assignment. It needs a little more simplification. P9	Lack of time	4 (5)	I am in a tough bind in the busy schedule. The patients are accepted in order, I miss that. The patients are waiting and their everything is ready. We have to think about them...They made an appointment and it’s the natural one. There are patient relatives came from Germany and you cannot postpone them. I don’t even want to take administrative function. Binds are bad. P26

Lack of Time

Lack of time was the top barrier for the participants of case 1 (11 of 14). Only four participants of case 2 reported time-related restrictions as a significant barrier. However, more than half of all participants reported lack of time as an important barrier. Ten participants mentioned difficulty in following up to complete online learning tasks. Some participants reported feeling overwhelmed by their requirements to complete all learning tasks due to their clinical and teaching workload.

Participants reported that allocating time to attend the courses was challenging in a busy workplace environment. They reported that advance arrangements should be made according to the course program. Some participants speculated that the long duration of the courses might adversely affect the face-to-face session attendance.

Beliefs and Assumptions

Beliefs and assumptions were a significant barrier in a BL faculty development program; it was the top barrier for participants in case 2 and the second most common barrier for participants in case 1. Integrating online components in which participants are used to taking courses by traditional methods of teaching and learning made different impressions on the faculty. Half of the participants mentioned that beliefs and assumptions, precisely age, change, and culture, affected their views.

Case 2 faculty reported that age might cause lack of concentration, lack of technology usage, and lack of ability to adapt. An altered routine was seen as another barrier. However, case 1 participants did not mention anything about the change.

Four of the participants mentioned culture as a barrier. The participants stated that getting used to one method and then adding other methods to learning may not help them. Moreover, the traditional point of view is also affected. They are afraid that introducing BL may bring about additional workload, especially learning tasks to be completed beforehand.

Online Learning Abilities

Online learning abilities were the final notable theme regarding barriers. Generational differences may create a gap in information and communication technology (ICT) skills. Eight participants stated that technology frightens some of the faculty. Furthermore, learning how to use such technology must be adopted and disseminated to their medical students. The more they feel "into" the online technology, the more they can overcome this perceived barrier to their online skills.

## Discussion

In this study, we sought to identify faculty members’ perceived enablers and barriers in a BL faculty development program. This study identified three barriers and eight enablers in the BL training program. BL program participation requires a level of technical and device familiarity. The use of technology and adapting to new methodologies in teaching may be overwhelming for some faculty. Beliefs and assumptions such as age, change, and culture were significant barriers for junior and senior faculty. There is a hesitancy around unexpected new practices, with faculty resisting the implementation of new methods, which may bring about new or additional responsibilities. Respondents were wary of committing to new methods while dealing with an overfull workload.

Similar to Wearne et al. [6], we found that core themes emerged, despite recruiting a heterogeneous participant group. We intentionally included junior and senior faculty but found common facilitators and barriers for both groups. Access and usage of the online components of BL programming were not universally favored by faculty. ICT skills and technology acceptance may impede the use of a BL approach to faculty development [6,17]. Our findings are similar to that of prior work. Computer literacy skills were also reported as a barrier to online learning by Lawn et al. [18]. Anshu et al. [19] mentioned that technical terminology might discourage faculty from actively participating in a BL faculty development program.

The faculty reported that workload-related lack of time kept them from attending and following up on activities in the BL program. A busy work life within a medical faculty environment was a major barrier, and the courses were seen as additional workload and were therefore not prioritized [20] compared to other duties for which the faculty are responsible. Our study participants were not the only subjects to report such concerns; lack of time, workload, isolation because of technology, and increases in student numbers have also been reported in the literature [20,21].

Addressing participant beliefs and assumptions upfront may mitigate some difficulties in adapting to a technology-forward BL experience. By emphasizing the incentives and aspects that facilitate successful learning experiences, these barriers may be mitigated by the inherent elements that we identified as enablers in the BL approach.

A decrease in the time required for face-to-face sessions was a good advantage of BL noted by the faculty in our study. Other studies reported a similar time constraint problem [2,17,20,22], and this study shows that BL reduces the time commitment necessary for face-to-face encounters.

Junior and senior faculty perceived personal development and improving teaching skills as significant enablers in the BL faculty development program. Pernar et al. described a faculty development program based on weekly emails with compact content to improve teaching skills that failed due to a lack of instructional design [23]. However, in a carefully designed program, faculty can establish a level of involvement that improves their teaching skills [24].

Our findings suggest that certification as an enticement enabled the junior faculty as they did not already have such a certificate, and they perceived certification to advance their academic careers. Indeed, the program's participants become certified after completing the faculty development program, and some program participants are required to obtain certification for career promotion (e.g., attending physicians). Vaughan et al. and Welch similarly reported that 10 weeks of teaching certification is required in Sweden to gain tenure [25,26]. Given the participants' inherent desire to improve their academic rank and the requirement for certification, it is not surprising that junior faculty perceived this aspect as an enabling element in a BL program.

The perceived quality of a course is a factor in the level of participation [20]. The novelty of BL and the application of new methods in their education provide faculty a sense of the future of educational programming and modalities. Shah et al. found that BL motivates learners for mentor support [27].

In a study by Fox et al., participants reported a sense of isolation in online learning programs without a supporting face-to-face encounter [28]. By providing face-to-face sessions and supporting courses with online content in the present study, faculty perceived BL was engaging. Participants felt that socializing in face-to-face sessions and discussing with and observing other participants online were enjoyable and facilitated peer learning.

A BL learning approach carries intrinsic incentives, but study participants also suggested that extrinsic rewards may play a role in the program's success. A system such as financial or other forms of rewards may entice faculty participation despite the time and workload barriers. Honoraria or an award for participation may foster motivation for future course participants [29].

During the coronavirus disease pandemic 2019, faculty had first-hand experiences of online interactions. This experience is expected to affect the future of faculty development and health professions education [30]. Faculty may expect an online component for any learning activity. As described in this study, BL will be imperative for faculty development.

Limitations

This study was conducted at a single center in Turkey, which may limit its transferability. As a qualitative study and single-center data source, one should not generalize the results. However, the diverse specialty of the participants may inform broader context in faculty development in medical sciences.

## Conclusions

BL can be used to design future faculty development programs. Faculty developers can utilize BL to foster engagement and motivate faculty for increased participation. While faculty seniority may affect use of the online tools, intuitive online platforms should be selected for wide acceptance among faculty. Online activities in faculty development should be approached cautiously. Workload and time management are main barriers in BL faculty development. Strategies to eliminate workload and provide guidance on time management are required for both junior and senior faculty. Goal setting for online activities should be aligned with the faculty’s priorities and the expectations from faculty development. The activities and the content should complement face-to-face sessions and provide opportunities for faculty to support their online learning skills.
